# Supplementary material for: Discovering the Ultimate Limits of Protein Secondary Structure Prediction
Source: Biomolecules. 2021 Nov 3;11(11):1627. doi: 10.3390/biom11111627 (PMC8615938; doi:10.3390/biom11111627)
Supplement: Supplementary file 1 [file biomolecules-11-01627-s001.zip › Data_S1.pdf]

Data S1. Additional supporting data

The estimated SSP upper limits for various secondary structure elements

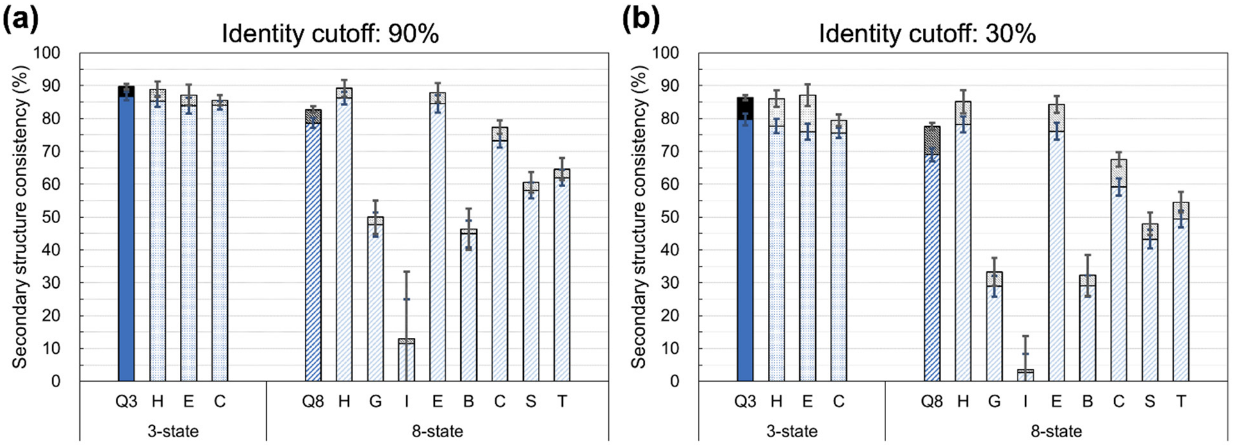

Figure DS1-1. Estimated upper limits of SSP for three- and eight-state SSEs. (**a**) The secondary structural consistency for homologs sharing <90% identities. (**b**) The consistency for homologs sharing <30% identities. In this study, most experiments and tests were repeated ten times by random sampling. In the present test, because of the low occurrence of the 8-state SSE I (π-helix), the SCOP-2.07 dataset was randomly divided into five subsets, meaning that the test was repeated five times. In every section of this figure, the first bar (estimated upper limits of Q3 and Q8) were adapted from Figure 6 for reference; those estimates were obtained by tenfold random sampling instead of fivefold. The raw data of this test are available in Table S7.

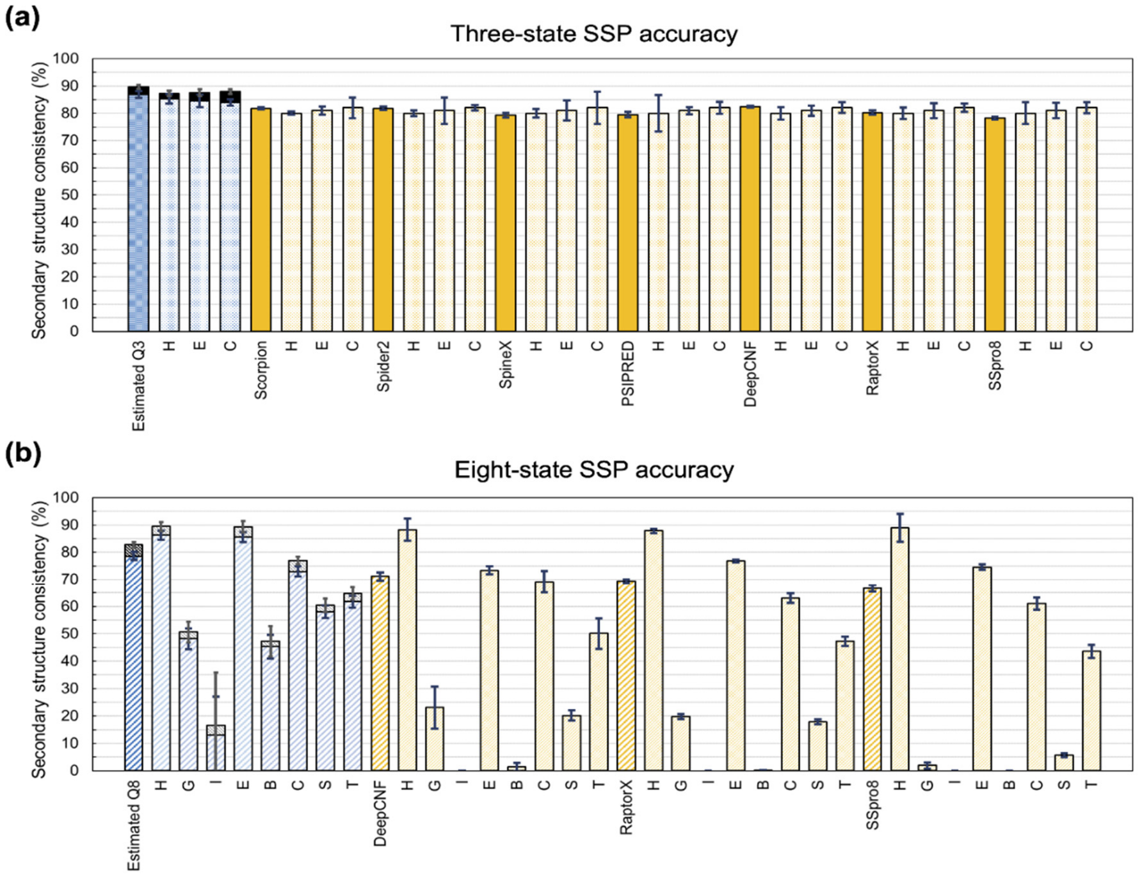

Figure DS1-2. Accuracies of state-of-the-art SSP methods evaluated with datasets sharing <90% sequence identities. (**a**) Three-state SSP accuracy. (**b**) Eight-state SSP accuracy. In each panel, the blue bars on the left indicate the estimated upper limits of Q3 and Q8 (the darker blue bars; obtained from Figure 6) and the estimated SSP accuracy for various SSEs (the lighter blue bars; obtained from Figure DS1-1).

The estimated SSP upper limits for residues with high or low solvent accessibility

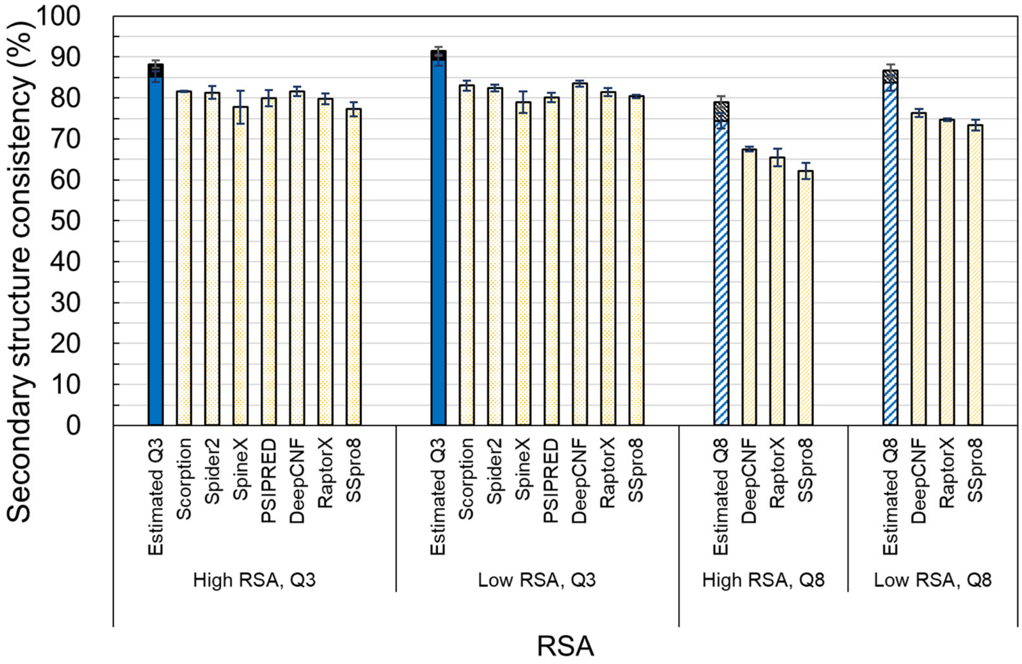

Figure DS1-3. Estimated upper limits of SSP and accuracies of state-of-the-art SSP methods for residues with different levels of solvent accessibility. In each section of this figure, the blue bar on the left exhibits the secondary structural consistency for residues of high or low relative solvent accessibility (RSA) between homologs sharing <90% identities. The seven SSP methods were evaluated with datasets sharing <90% sequence identities.

The estimated SSP upper limits for residues with high or low B-factors

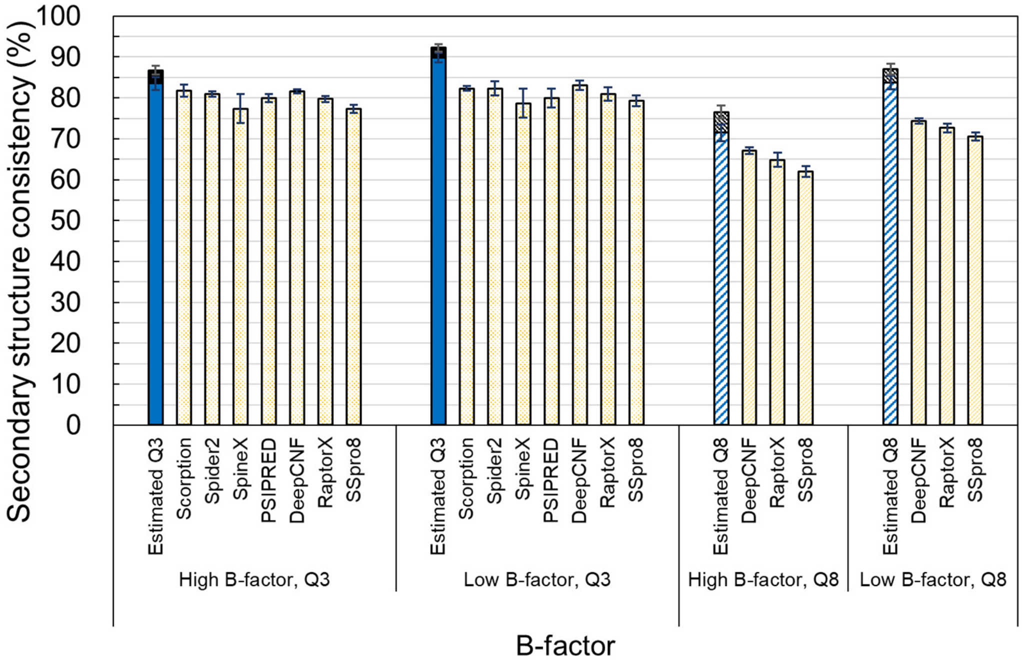

Figure DS1-4. Estimated upper limits of SSP and accuracies of state-of-the-art SSP methods for residues with different levels of B-factor. In each section of this figure, the blue bar on the left exhibits the secondary structural consistency for residues of high or low normalized B-factors (Z-score cutoff: 0.03) between homologs sharing <90% identities. The seven SSP methods were evaluated with datasets sharing <90% sequence identities.
