# Supplementary material for: Discovering the Ultimate Limits of Protein Secondary Structure Prediction
Source: Biomolecules. 2021 Nov 3;11(11):1627. doi: 10.3390/biom11111627 (PMC8615938; doi:10.3390/biom11111627)
Supplement: Supplementary file 1 [file biomolecules-11-01627-s001.zip › Figure_S1.pdf]

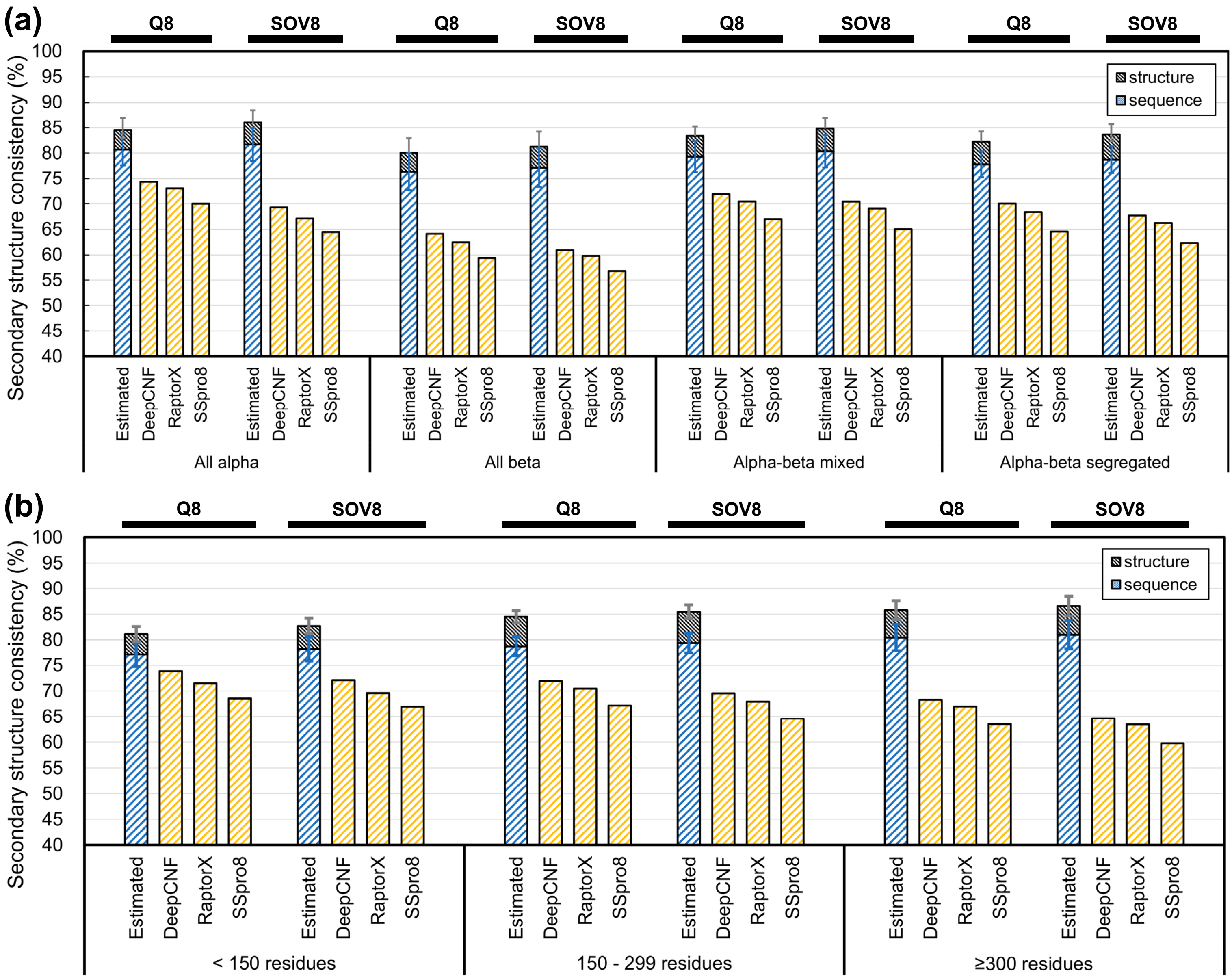

Figure S1. The eight-state prediction accuracy of state-of-the-art SSP methods for proteins of different structural classes or sizes. (**a**) The accuracies for proteins of different structural classes. (**b**) The accuracies for proteins of different sizes. In these charts, the upper limit of SSP accuracy estimated by sequence and structural alignments at 90% sequence identity cutoff are respectively indicated by the blue and black bars. The same tendencies among different class/size groups revealed by these 8-state prediction results were also observed in 3-state predictions. See Figure 8 and the main text for details.
