# Supplementary figures and images for: Discovering the Ultimate Limits of Protein Secondary Structure Prediction
Source: Biomolecules. 2021 Nov 3;11(11):1627. doi: 10.3390/biom11111627 (PMC8615938; doi:10.3390/biom11111627)

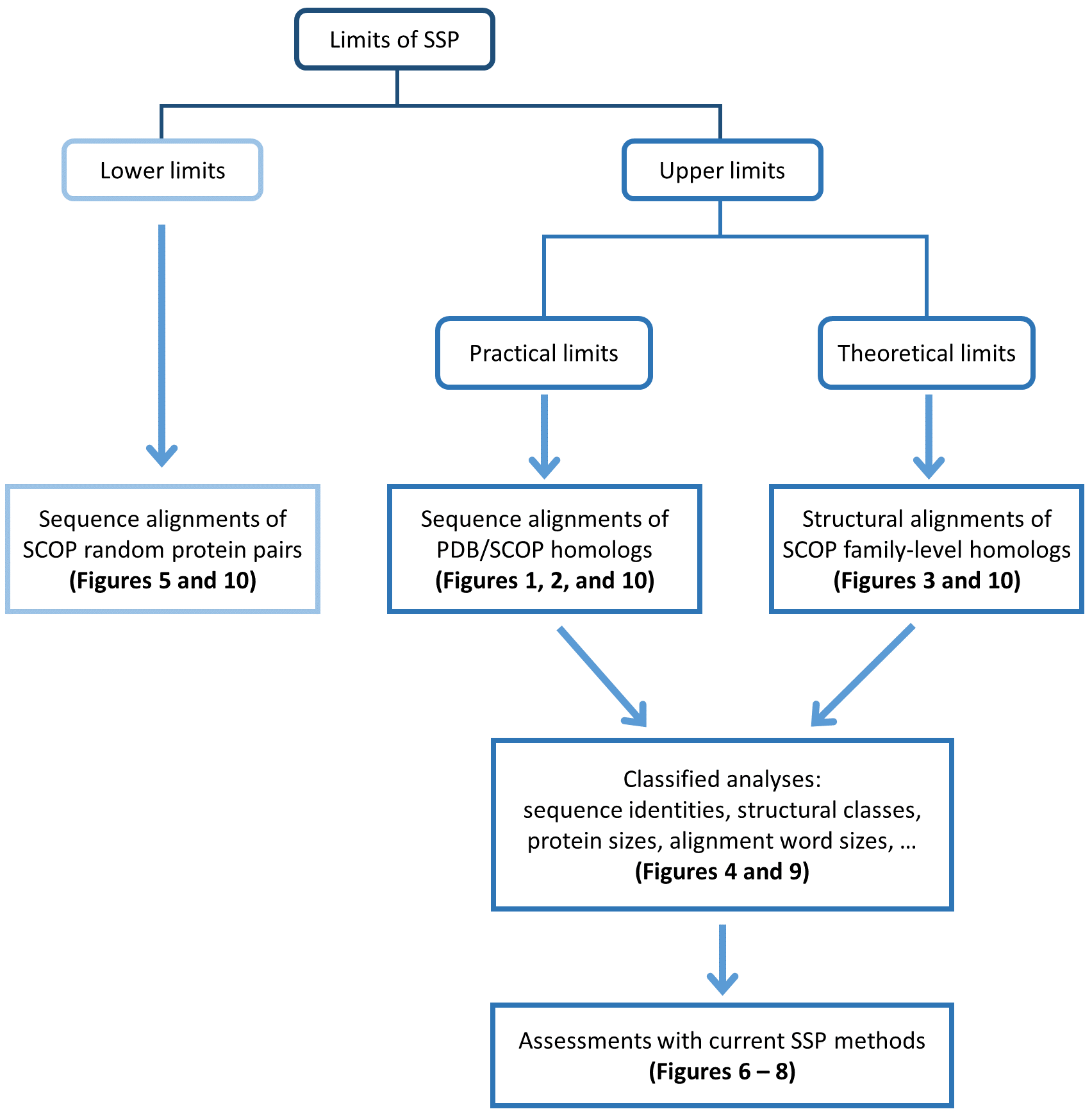

Supplement: Supplementary file 1 [file biomolecules-11-01627-s001.zip › Figure_A1.png]
